# RHAMM deficiency disrupts folliculogenesis resulting in female hypofertility

**DOI:** 10.1242/bio.201410892

**Published:** 2015-03-06

**Authors:** Huaibiao Li, Jürgen Moll, Anne Winkler, Lucien Frappart, Stéphane Brunet, Jana Hamann, Torsten Kroll, Marie-Hélène Verlhac, Heike Heuer, Peter Herrlich, Aspasia Ploubidou

**Affiliations:** 1 Leibniz Institute for Age Research – Fritz Lipmann Institute, Beutenbergstrasse 11, D-07745 Jena, Germany; 2 Forschungszentrum Karlsruhe, Institut für Toxicologie und Genetik, Postfach 3640, D-76021 Karlsruhe, Germany; 3 INSERM, Oncogenèse et Progression Tumorale, Université Claude Bernard Lyon I, 28 rue Laënnec, 69373 Lyon, France; 4 Collège de France, 11 place Marcelin Berthelot, 75231 Paris, France; 5 Leibniz Research Institute for Environmental Medicine (IUF), 40021 Düsseldorf, Germany; 6 Present address: Boehringer-Ingelheim RCV and Co KG, Dr. Boehringer-Gasse 5-11, A-1121 Vienna, Austria.; 7 Present address: Georg-August-University Göttingen, Dept. of Neuropathology, Robert-Koch-Strasse 40, D-37075 Göttingen, Germany.

**Keywords:** Gametogenesis, Folliculogenesis, Hypofertility, Spindle, RHAMM, Oriented mitosis, Centrosome

## Abstract

The postnatal mammalian ovary contains the primary follicles, each comprising an immature oocyte surrounded by a layer of somatic granulosa cells. Oocytes reach meiotic and developmental competence via folliculogenesis. During this process, the granulosa cells proliferate massively around the oocyte, form an extensive extracellular matrix (ECM) and differentiate into cumulus cells. As the ECM component hyaluronic acid (HA) is thought to form the backbone of the oocyte-granulosa cell complex, we deleted the relevant domain of the *Receptor for HA Mediated Motility* (RHAMM) gene in the mouse. This resulted in folliculogenesis defects and female hypofertility, although HA-induced signalling was not affected. We report that wild-type RHAMM localises at the mitotic spindle of granulosa cells, surrounding the oocyte. Deletion of the RHAMM C-terminus *in vivo* abolishes its spindle association, resulting in impaired spindle orientation in the dividing granulosa cells, folliculogenesis defects and subsequent female hypofertility. These data reveal the first identified physiological function for RHAMM, during oogenesis, and the importance of this spindle-associated function for female fertility.

## INTRODUCTION

The postnatal mammalian ovary contains primary oocytes, each enclosed in a single layer of somatic granulosa cells, forming the so-called primordial ovarian follicles (Pepling and Spradling, 2001). These immature oocytes, which are arrested in prophase of Meiosis I (Eppig, 1993), acquire meiotic and developmental competence during folliculogenesis, i.e. the transition from the small primordial follicle to the large multilayered pre-ovulatory follicle. During this process, the oocyte progressively grows while the granulosa cells proliferate and differentiate (reviewed in Li and Albertini, 2013).

Folliculogenesis requires oocyte-granulosa cell interaction, which is stabilised by extracellular matrix (ECM) components and, in its last two stages, depends also on hormones. Following activation, the primordial follicles undergo four successive stages of development, defined by oocyte growth and by the number and type of cells surrounding the oocyte (Hoage and Cameron, 1976; reviewed by Pepling, 2006): accompanied by massive expansion of the granulosa cell layer, the primordial follicles give rise to (i) primary follicles which in turn progress to (ii) secondary follicles. Stimulated by gonadotropins (FSH and LH), further proliferation of the granulosa cells gives rise to the (iii) multi-layered antral follicle containing antral cavities (Messinis et al., 2010). In the final stage of folliculogenesis, the granulosa cells differentiate into mural and cumulus cells (the latter surrounding the oocyte), a single large antral cavity is formed and the oocyte reaches its final growth in the so-called (iv) preovulatory follicle, ready for ovulation. The fully-grown oocyte is tightly enclosed into several layers of thousands of cumulus cells, held together by gap junctions and stabilized by extensive ECM. This is called the cumulus-oocyte complex (reviewed by Russell and Salustri, 2006). The cumulus-oocyte association is retained through ovulation, whereupon the follicle wall ruptures releasing the complex.

Granulosa cell proliferation and multilayered cell complex formation around the maturing oocyte and its ECM components, are essential features of female germ cell maturation. Distinct changes in the morphology of the proliferating granulosa cells and the precise spatial orchestration of their mitotic divisions, give rise to the multi-layered follicle (Da Silva-Buttkus et al., 2008). However, only few of the stimulating and inhibitory regulatory molecules, which obviously accompany this complex process, are known (e.g. Fan et al., 2008; Fan et al., 2009; reviewed by Sánchez and Smitz, 2012).

A significant portion of the ECM of the follicles consists of hyaluronan (or hyaluronic acid, HA). Both oocytes and cumulus cells produce HA during folliculogenesis (Salustri et al., 1992; Ueno et al., 2009). There is yet no functional evidence that HA is essential for folliculogenesis, although the co-expression and the time courses of hyaluronan synthases and hyaluronan binding surface proteins suggest such a role (Ohta et al., 1999; Schoenfelder and Einspanier, 2003). HA could simply serve as a structural component or it could induce a signalling cascade. For both functions, cells must possess one or several hyaluronan-binding receptors. Two major surface receptors for HA have been reported, CD44 and RHAMM (Hardwick et al., 1992; Hofmann et al., 1998a; Lesley et al., 1992; Yoneda et al., 1990); the latter's HA receptor function has also been disputed (Hofmann et al., 1998b; Jiang et al., 2013).

For both receptors, links to extracellular signalling have been reported. However the physiological and developmental functions of RHAMM are unknown. In cell models, RHAMM has been reported to mediate HA-induced ERK1/2 pathway activation and cell migration (Hardwick et al., 1992, Hamilton et al., 2007). Apart from these putative extracellular activities, RHAMM functions in centrosomal and acentrosomal spindle assembly (Maxwell et al., 2003; Groen et al., 2004) as well as in spindle orientation (Dunsch et al., 2012) have been documented in cell extracts and cultured cells.

To explore whether RHAMM is required for gametogenesis, we generated a deletion of the C-terminal putative HA-binding domain as well as the centrosome-targeting sequence of the mouse RHAMM gene (*hmmr*). The mutant animals are viable, but the RHAMM truncation resulted in female hypofertility. We report here the underlying mechanism, which reveals the first physiological RHAMM function and, in addition, demonstrates the requirement for oriented cell divisions in female gametogenesis.

## RESULTS

### RHAMM is expressed in the female reproductive organs

**Fig. 1.**
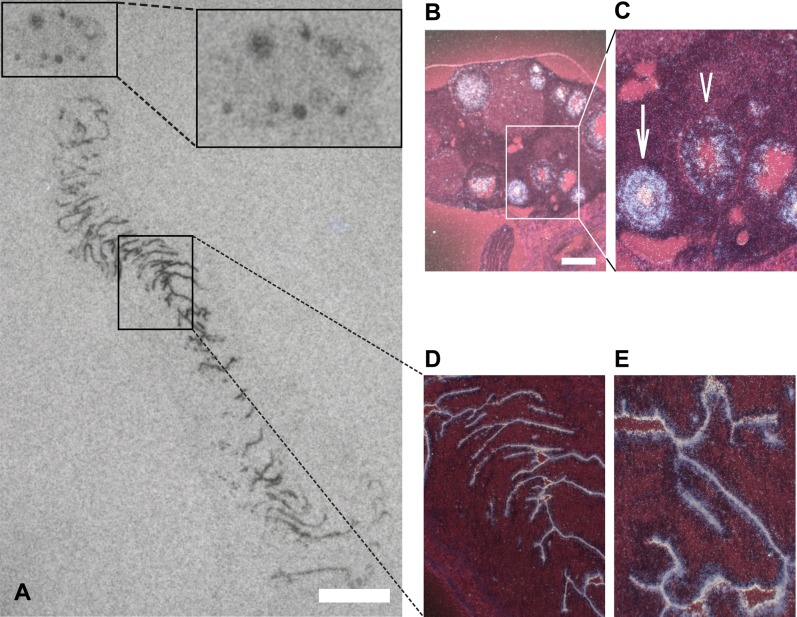
RHAMM mRNA expression and localization in the mouse ovary and uterus. (A) Representative section of the female reproductive organs, subjected to radioactive *in situ* hybridization, reveals strong expression of RHAMM along the (mitotic) epithelium of the uterus glands and in ovarian follicles of different maturation stages, visualised on an X-ray film autoradiogram. (B,C) RHAMM expression in the ovarian follicles is restricted to the proliferative granulosa cells (GCs) surrounding the oocyte, as illustrated by dark-field illumination of Cresyl-violet counterstained ovary sections. Consistent with this, the highest expression, as indicated by labelling intensity, is observed in secondary follicles containing highly proliferative GCs (arrow) and it is decreased in antral follicles (arrowhead) concomitantly with the decreased proliferation of GCs in these follicles. (D,E) Magnification showing the labelling along the epithelium of the uterus glands. Scale bars: 5 mm (A); 5 µm (B,D).

RHAMM expression had been detected previously in mRNA extracted from cattle cumulus-oocyte complexes (Schoenfelder and Einspanier, 2003), but its cell specific expression and localization in the reproductive system was unknown. We therefore analyzed RHAMM mRNA expression in the ovary and uterus of wild type mice by radioactive *in situ* hybridization. RHAMM mRNA was highly expressed along the mitotic epithelium of the uterus, as well as in distinct foci within the ovary that correspond to the ovarian follicles (Fig. 1A). RHAMM expression in the follicles was restricted to the proliferative granulosa cells surrounding the oocyte (Fig. 1B,C). The highest transcript levels were observed in secondary follicles containing highly proliferative granulosa cells (Fig. 1C, arrow) whereas lower hybridization signal intensities were found in follicles with growing antrum (Fig. 1C, arrowhead), characterised by decreased granulosa cell proliferation.

### The *hmmr^m/m^* mouse expresses C-terminus truncated RHAMM, devoid of the centrosome-targeting and HA-binding protein domains

**Fig. 2.**
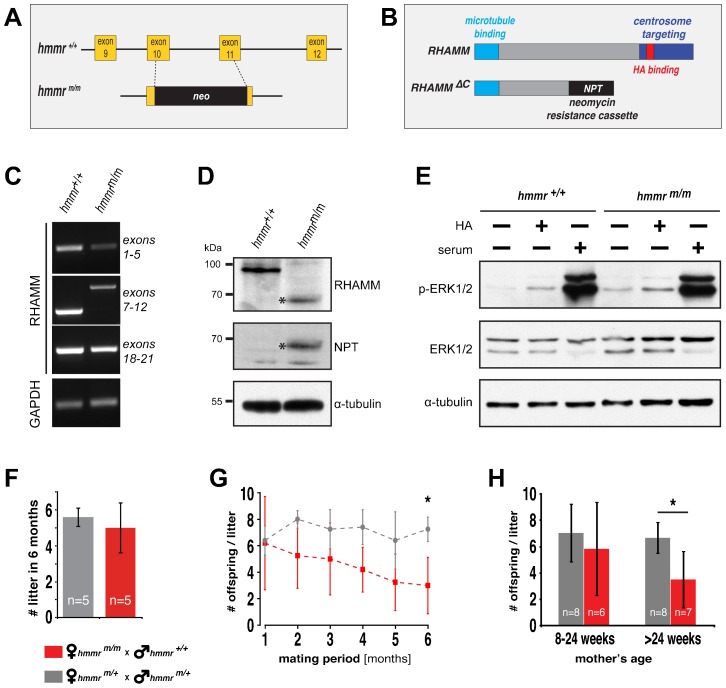
Deletion of the RHAMM C-terminus results in expression of a truncated protein variant and consequent female hypofertility, but it does not prevent HA-induced signalling. (A) Schematic representation of the strategy employed in the generation of the *hmmr^m/m^* mouse. A neomycin-resistance cassette (neo) with an in-frame stop codon was targeted into the genomic sequence of wild type *hmmr^+/+^*, replacing part of exons 10 and 11 as well as the intronic region. (B) The resulting premature translation stop gave rise to a truncated (RHAMM ΔC) protein, lacking the HA-binding and centrosome-targeting domain of wild type RHAMM, and fused to the neomycin-resistance gene product neomycin phosphotransferase (NPT). (C) RT-PCR analysis, employing primer pairs targeting different RHAMM genomic regions, demonstrates the insertion of the neomycin cassette, as indicated by the increased size of the PCR gene product in the *hmmr^m/m^* sample, in the region of exons 7–12. GAPDH was used as control. (D) The full length (95 kDa) RHAMM protein is expressed in *hmmr^+/+^* MEFs, while their mutated *hmmr^m/m^* counterparts express a truncated 65 kDa protein (asterisk) fused to the NPT gene product, as demonstrated by the western blot probed with anti-RHAMM and anti-NPT antibodies. α-tubulin was used as loading control. (E) Deletion of the RHAMM C-terminus does not prevent HA-induced ERK1/2 activation, as indicated by the level of phosphorylated ERK1/2 (p-ERK1/2) in *hmmr^m/m^* MEFs compared to wild type *hmmr^+/+^* controls, assessed by western blotting. ERK1/2 and α-tubulin were used as loading controls. (F,G) Average number of litters (F) and litter size (G) (denoted by the average number of offspring per litter), produced by mating pairs of the indicated genotype over a period of 6 months. The mice used in the breeding assay were 8–12 weeks old. (H) Age-dependent female hypofertility, induced by the RHAMM deficiency, is indicated by the reduction in litter size of *hmmr^m/m^* females older than 24 weeks. A minimum of 25 litters per genotype and time point was quantified. In F,G,H, data are presented as mean±s.d.; n indicates the number of mating pairs per group, *p<0.05.

The functions of RHAMM, thus far described in cultured cells, are mediated by its centrosome-targeting and HA-binding domains, which are located in the C-terminus (Fig. 2B) and are responsible for the spindle assembly and cell migration roles of the protein, respectively. To inactivate this region in the mouse, a neomycin cassette with in-frame stop codons was inserted in the mouse genome, replacing a region between exon 10 and 11 of hmmr (Fig. 2A).

RT-PCR analysis of RHAMM mRNA expression, in mouse embryonic fibroblasts (MEF) derived from the resulting mutant animals (*hmmr^m/m^*), showed that insertion of the neomycin resistance cassette did not prevent mRNA expression of the RHAMM C-terminus (Fig. 2C). However, the premature stop-codon in the neomycin cassette resulted in translation of a truncated protein containing the neomycin-resistance gene product and with the expected molecular weight of 65 kDa, expressed in *hmmr^m/m^* mutant cells (Fig. 2D). A ∼95 kDa protein was detected in wild type samples (*hmmr^+/+^*), consistent with the predicted molecular weight of the full-length RHAMM protein (Fig. 2D). An antibody raised against a RHAMM N-terminus peptide, thus cross-reacting with both the full-length as well as the C-terminus truncated protein, was used for immunoblotting analysis.

### The RHAMM mutant mice are viable

The homozygous *hmmr^m/m^* mice were viable and normal in appearance. In view of the role of RHAMM in spindle assembly described *in vitro*, the viability was a surprise. It indicates that the centrosome-targeting function is not essential for mitotic spindle assembly. Also unexpected was that absence of the RHAMM HA-binding motif did not interfere with viability, as HA has been implicated in many cellular processes (Sherman et al., 1994; Toole, 2004). We therefore explored whether HA-mediated signalling is compromised in the *hmmr^m/m^* background.

### The HA-binding domain of RHAMM is dispensable for HA-induced ERK1/2 activation

Because RHAMM was originally identified as mediator of HA-induced ERK1/2 activation (Zhang et al., 1998), we examined whether deletion of the RHAMM HA-binding domain prevents HA-induced ERK1/2 phosphorylation. Wild type and RHAMM mutant MEF were stimulated with high molecular weight HA and the phosphorylated ERK1/2 (p-ERK1/2) protein level was subsequently determined by western blotting. As expected, HA stimulation increased p-ERK1/2 level in *hmmr^+/+^* MEF. Surprisingly, however, p-ERK1/2 level was also increased in *hmmr^m/m^* cells (Fig. 2E), indicating that deletion of the RHAMM HA-binding domain is dispensable for HA-induced ERK1/2 phosphorylation. Apparently, HA stimulates ERK1/2 phosphorylation through signalling receptors other than RHAMM.

### The *hmmr^m/m^* female mice display age-dependent hypofertility

Mating of *hmmr^m/m^* females with wild type *hmmr^+/+^* male mice revealed an age-dependent hypofertility of the mutant females (Fig. 2G,H). As haplodeficiency of *hmmr* did not induce fertility defects, *hmmr^+/m^* mating pairs were used as controls. There was no significant difference between *hmmr^m/m^* and *hmmr^+/m^* females in the total number of litter born over a period of 6 months (Fig. 2F), suggesting that the pregnancy of *hmmr^m/m^* females was normal. However, analysis of the litter size revealed a progressive decrease in the number of offspring/litter of young (8–24 week old) *hmmr^m/m^* females when compared to controls, which became significant in older animals (>24week old) (Fig. 2G,H). These findings indicate that deletion of the RHAMM C-terminus leads to age-dependent female hypofertility.

### RHAMM deficiency does not affect oocyte meiotic maturation

The oocytes reach meiotic and developmental competence during folliculogenesis (Sánchez and Smitz, 2012). After successful completion of folliculogenesis, oocytes resume meiosis and undergo meiotic divisions to produce the gamete awaiting fertilization. Success of meiotic division relies on the assembly of a functional acentrosomal spindle (Kaláb et al., 2011; Dumont et al., 2007). As RHAMM is critical for acentrosomal spindle assembly in *Xenopus* oocyte extracts (Groen et al., 2004; Joukov et al., 2006), we tested whether mouse female hypofertility, caused by the deletion of the RHAMM C-terminus, is due to acentrosomal spindle assembly defects, in oocyte meiosis.

To do this, mature prophase-arrested oocytes were isolated from 10 week-old mice and cultured *in vitro* (Brunet and Maro, 2007), in order to analyse the completion of meiotic maturation by time-lapse imaging (Brunet et al., 2008). Both *hmmr^+/+^* (76%) and *hmmr^m/m^* (71.4%) oocytes did extrude a polar body upon culture (M2, supplementary material Fig. S1B). In addition, the kinetics of germinal vesicle breakdown (GVBD) and 1^st^ polar body (PB1) expulsion were similar in wild type and mutant oocytes (supplementary material Fig. S1C,D), clearly showing that the RHAMM C-terminus deletion did not impair the meiotic maturation process of competent oocytes.

As the *hmmr^m/m^* females display age-dependent hypofertility (Fig. 2G,H), we also analyzed the meiotic maturation of oocytes of 26 week-old mice. Despite delays in GVBD and polar body extrusion kinetics, most of the oocytes reached Meiosis 2 (supplementary material Fig. S1F,G), indicating that, under these conditions, the meiotic maturation process was not significantly impaired.

Strikingly, in the 10-week-old females, the number of oocytes recovered from the *hmmr^m/m^* ovary was only a third of those recovered from the wild type ovary (*hmmr^m/m^* n = 10; *hmmr^+/+^* n = 30). Moreover, this discrepancy increased in the 26-week-old females (*hmmr^m/m^* n = 2, n = 2, n = 9 collected from 3 individuals and pooled; *hmmr^+/+^* n = 17). This observation suggested that the RHAMM truncation has affected the oogenesis process prior to the step of meiotic maturation.

### RHAMM deficiency impairs ovarian folliculogenesis

Analysis of the reproductive system of *hmmr^+/+^* females revealed no abnormalities in the cervix and uterus, despite the high level of RHAMM mRNA expression in the epithelium of the uterus in wild type animals. Since the number of oocytes recovered from the ovaries of *hmmr^m/m^* mice was approximately a third of those recovered from wild type ones, we asked whether defective oogenesis is the cause of the age-dependent hypofertility of the *hmmr^m/m^* females.

**Fig. 3.**
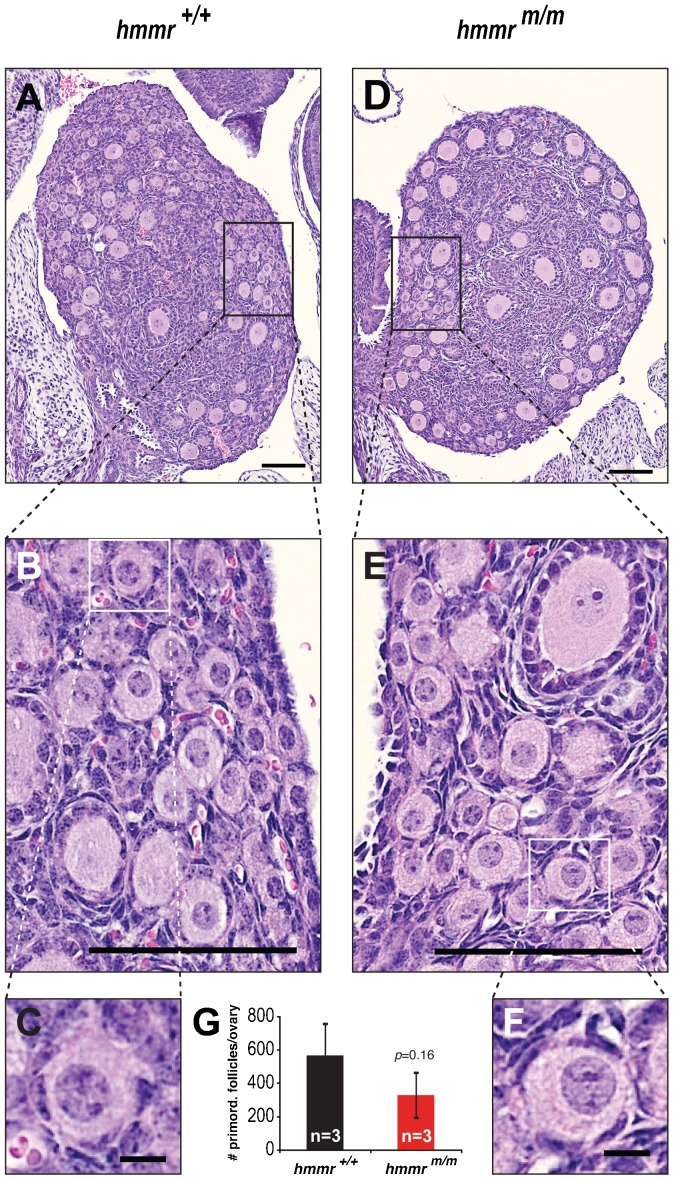
RHAMM deficiency has no adverse effects on primordial follicle formation. Visualization (A–F) and quantification (G) of primordial follicles in mouse ovaries, at post-natal day 7. (A–F) Hematoxylin- and eosin-stained representative sections of ovaries from *hmmr^+/+^* (A) and *hmmr^m/m^* (D) females. Boxes indicate the magnified areas. No morphological defects were observed in *hmmr^m/m^* follicles (D–F) as compared to controls (A–C). (G) Quantification of primordial follicles, in (one every five) 4-µm-thick serial sections of ovaries of *hmmr^+/+^* (n = 3) and *hmmr^m/m^* (n = 3) mice, showed no statistically significant difference in the number of these follicles between the genotypes. Data presented as mean±s.d. Scale bars: 100 µm (A,B,D,E); 10 µm (C,F).

In order to determine whether the *hmmr^m/m^* ovaries have a diminished reservoir of oocytes, the ovaries of mice at post-natal day 7 (PND7) were analyzed (Fig. 3). At this time point, primordial follicles' formation has been completed and the total number of oocytes available for reproduction is fixed (Pepling and Spradling, 2001; Pepling, 2006). The primordial follicles in *hmmr^m/m^* ovaries appear morphologically normal (Fig. 3D–F). Their quantification (Fig. 3G) indicated a potentially reduced reservoir of oocytes in *hmmr^m/m^* mutants but no statistically significant difference in the number of these follicles between mutant and wild-type animals.

**Fig. 4.**
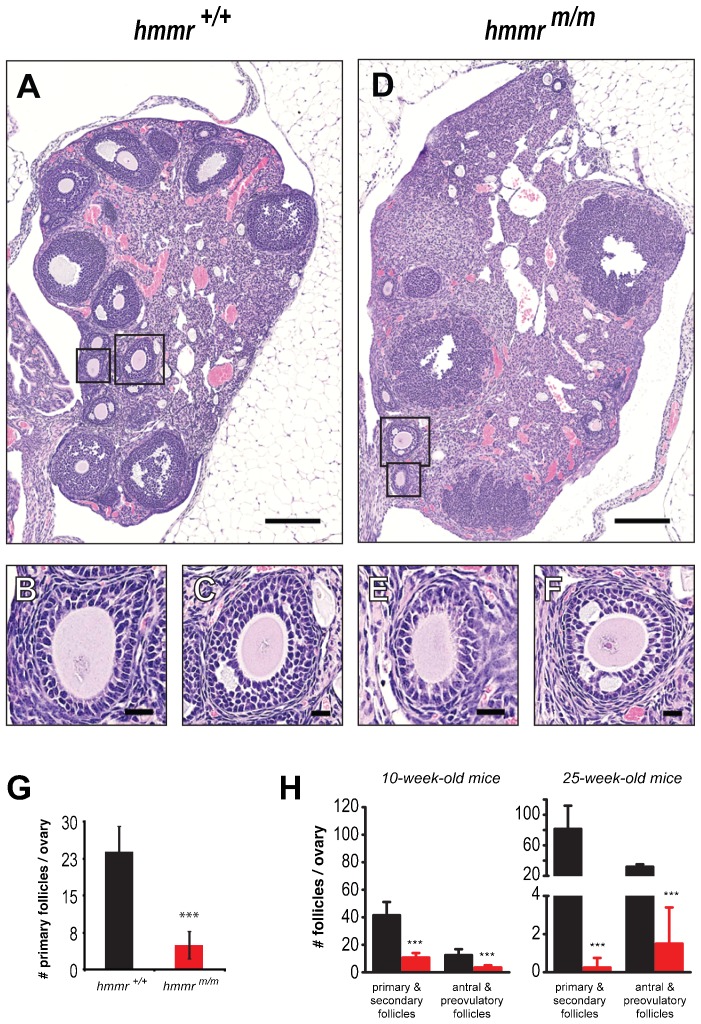
The RHAMM centrosome-targeting domain is required for adult ovarian folliculogenesis. Visualization (A–F) and quantification (G,H) of ovarian follicles in adult mice reveals a severe folliculogenesis defect in *hmmr^m/m^* mutants. (A–F) Hematoxylin- and eosin-stained representative sections of ovaries from *hmmr^+/+^* (A) and *hmmr^m/m^* (D) females demonstrate depletion of the *hmmr^m/m^* ovary of follicles. However, the few follicles formed in *hmmr^m/m^* ovaries (E,F) exhibit no morphological defects as compared to controls (B,C). Boxes in A and D indicate the magnified areas in B,C and E,F, respectively. (G) Quantification of primary follicles (e.g. B,E) in ovaries of 10-week-old mice, reveals a severe folliculogenesis defect in *hmmr^m/m^* mutants, which contain up to 5-fold decreased number of these follicles when compared to their wild type counterparts. (H) The defective folliculogenesis in RHAMM mutants is further demonstrated by the significant reduction of both immature (primary, secondary) and mature follicles (antral, preovulatory) in *hmmr^m/m^* ovaries, in 10-week-old as well as in 25-week-old mice. In G,H (10-week-olds), *hmmr^+/+^* n = 11 and *hmmr^m/m^* n = 4; in H (24-week-olds), *hmmr^+/+^* n = 4 and *hmmr^m/m^* n = 4. Data presented as mean±s.d.; ***p<0.001. Scale bars: 200 µm (A,D); 20 µm (B,C,E,F).

In ovaries of 10-week-old *hmmr^m/m^* mice, the number of primary follicles was decreased 5-fold as compared to the controls (Fig. 4G) indicating a defect in the transition from primordial to primary follicles. Moreover, in *hmmr^+/+^* ovaries, follicles of various maturation stages were present, indicating ongoing folliculogenesis (Fig. 4A). In contrast, mostly degenerative (“atretic”) follicles, with increased interstitial tissue, can be seen in a representative section of an *hmmr^m/m^* ovary (Fig. 4D). Quantification of the ovarian follicles in adult animals, using (one in every five) 4 µm semi-serial sections of complete ovaries (Canning et al., 2003; Tilly, 2003) from 10- and 25-week-old females, confirmed these observations (Fig. 4H). The number of both immature (primary and secondary) and mature follicles (antral and preovulatory) was very significantly decreased in *hmmr^m/m^* ovaries, compared to their *hmmr^+/+^* counterparts, at both age time-points analyzed. As mice got older, the number of immature and mature follicles increased in the *hmmr^+/+^* ovaries while decreasing (up to 100-fold) in *hmmr^m/m^* ones (Fig. 4H).

In conclusion, the *hmmr^m/m^* adult female mice exhibit a very significant reduction of ovarian follicles, indicating that the RHAMM C-terminus deletion impairs folliculogenesis, eventually reducing the pool of fully mature oocytes available for fertilization.

### The RHAMM C-terminus localises the protein at the mitotic spindle of granulosa cells

**Fig. 5.**
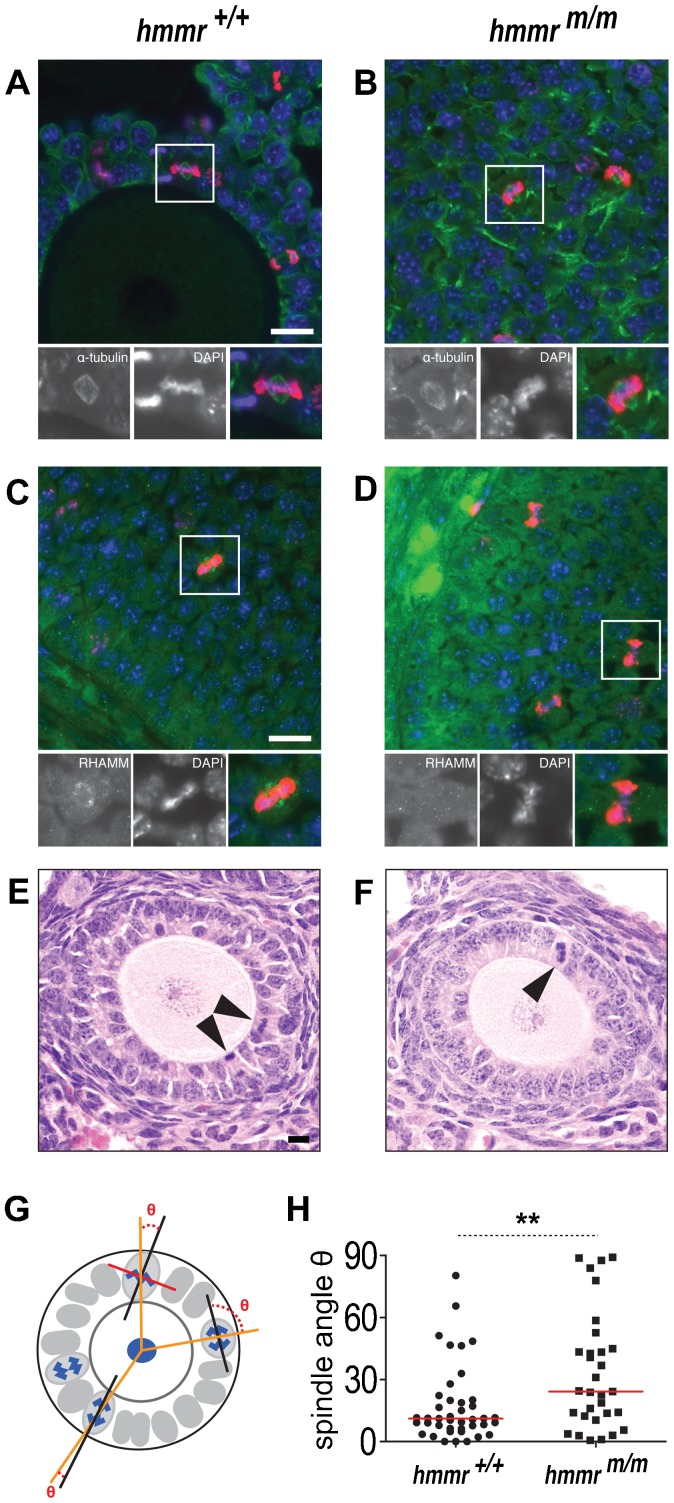
The RHAMM centrosome-targeting domain is required for RHAMM association with the mitotic spindle of the granulosa cells, where it regulates the orientation of the spindle axis. (A–D) RHAMM is localised at the mitotic spindle of granulosa cells, in follicles of *hmmr^+/+^* mice (C). This localization is abolished by the deletion of the RHAMM centrosome targeting domain in the *hmmr^m/m^* mutants (D), as shown in sections of ovaries labelled with anti-RHAMM (green) (C,D) and anti-pH3 (red) antibodies plus DAPI (blue) (A–D). No obvious spindle defects were observed in the granulosa cells of *hmmr^m/m^* mutants (B); microtubules were labelled with anti-α-tubulin antibody (green) (A,B). In A–D, the framed mitotic cells are magnified in the panels below each main image, showing the indicated single- and the merged triple-immunofluorescence labeling. (E–H) Ovarian follicle sections from 10-week-old mice stained with hematoxylin and eosin to visualise the mitotic granulosa cells (arrowheads) (E,F). Follicles with 1–2 layers of granulosa cells were used in quantification of the angle θ between oocyte-basal membrane (orange line) and spindle axes (black line), as schematically illustrated in G (see also Materials and Methods). Granulosa cells in *hmmr^+/+^* ovaries orient their spindle axis parallel to the oocyte-granulosa cell axis, as indicated by the small θ angle (H) (median 11.1°). The orientation of division is impaired in *hmmr^m/m^* granulosa cells (H) (median 24.2°) which assemble bipolar spindles without RHAMM (B,D). The difference of the two populations is 11.7° with a 95% confidence interval. The follicles of 4 *hmmr^+/+^* (n = 41 mitotic cells) and 8 *hmmr^m/m^* (n = 31 mitotic cells) mouse ovaries were used in the quantification. Scale bar: 20 µm; **p<0.01; red lines in H indicate the median angle.

In cultured cells, RHAMM associates with the mitotic spindle (Assmann et al., 1999; Maxwell et al., 2003; Groen et al., 2004; Maxwell et al., 2011) via its C-terminal centrosome-targeting domain (Maxwell et al., 2003). Consistent with these data, RHAMM localised at the mitotic spindle of granulosa cells in wild type ovaries (Fig. 5C), while this localization was completely abolished in *hmmr^m/m^* granulosa cells (Fig. 5D). Our *in vivo* data, confirmed, therefore, the previous observations in cell culture experiments.

Furthermore, in both wild type and *hmmr^m/m^* ovaries, the spindle morphology in granulosa cells was normal (Fig. 5A,B), indicating a non-essential role of RHAMM in centrosomal spindle assembly *in vivo*.

### RHAMM regulates the oriented mitotic division of granulosa cells

During primary follicle development, granulosa cells have been shown to divide in an oriented manner, with the cell division axis perpendicular to the oocyte and basal lamina (Da Silva-Buttkus et al., 2008). This division geometry may promote the formation of the granulosa layers and it has been postulated to be important for folliculogenesis (Da Silva-Buttkus et al., 2008).

During mitosis, the cell division axis is dictated by the positioning of the spindle within the cell, which is perpendicular to the metaphase plate (reviewed by Bornens, 2008). We reasoned that the absence of RHAMM on the spindle of *hmmr^m/m^* granulosa cells could perturb spindle positioning and alter cell division geometry.

To test this hypothesis, we analyzed the orientation of granulosa cell division, in 10-week-old mice. Follicles with one or two layers of granulosa cells were selected (Fig. 5E,F) and the long spindle axis of granulosa cells at metaphase or anaphase was used to determine the cell division plane (Fig. 5G, black line). For each granulosa cell, the angle θ between this plane and the axis determined by the oocyte and granulosa cell centres (Fig. 5G, orange line) was measured (Fig. 5G). In wild type ovaries, as expected, the small θ (median 11.1°) indicated that granulosa cells orient their spindle axis parallel to the oocyte-granulosa cell axis and perpendicular to the oocyte surface, generating proximal and distal daughter cells.

In contrast, in the *hmmr^m/m^* granulosa cells, the average θ angle measured was significantly higher, due to a wider distribution of this parameter. This indicated that, in the mutant, spindle orientation was impaired (Fig. 5H). Strikingly, in some cases, spindles were even found positioned perpendicular to the long cell axis, reinforcing the idea that the spindle orientation is determined, in wild type granulosa cells, via the RHAMM activity. The Kolmogorov-Smirnov test showed that the distribution of spindle angles in the mutant cells does not differ from the random distribution (p = 0.6 for *hmmr^m/m^*), in stark contrast to the wild type in which spindle angles differ very significantly (p = 1.6×10^−11^ for *hmmr^+/+^*) from the random distribution.

Taken together these data indicate that RHAMM, via its centrosome targeting domain, associates with the spindle of granulosa cells, contributes to its positioning and, in turn, to the geometry of the granulosa cell division. The disoriented positioning of the spindle in the *hmmr^m/m^* cells most probably contributes to alter the formation of a multilayered and functional follicle.

## DISCUSSION

### RHAMM expression in the proliferating granulosa cells of the ovarian follicles is consistent with its mitotic upregulation

We report that RHAMM mRNA is expressed in the adult mouse ovary and localized in ovarian follicles containing highly proliferative granulosa cells, in particular in primary, secondary and pre-antral follicles. In cultured cells, RHAMM mRNA expression is known to be cell cycle regulated, reaching the highest level at G2/M (Sohr and Engeland, 2008). The mRNA labelling observed in uterus and ovarian follicles is therefore consistent with an upregulation of RHAMM expression in highly proliferative tissues.

In its reported (Hardwick et al., 1992; Yang et al., 1993) but also disputed (Hofmann et al., 1998b; Jiang et al., 2013) function as HA receptor, RHAMM would be expected to be expressed in regions of high HA synthesis. In the ovaries, HA is synthesized in the last stage of folliculogenesis, in pre-ovulatory follicles (Zhuo and Kimata, 2001), concomitantly with a surge of expression of its receptor, CD44 (Ohta et al., 1999; Schoenfelder and Einspanier, 2003; Yokoo et al., 2010). In contrast, RHAMM is expressed at steady state during cattle oocyte maturation (Schoenfelder and Einspanier, 2003) and, in the cell type specific localization reported here, the peak of RHAMM expression occurs in the highly expanding GCs of secondary follicles (Fig. 1). In light of the high proliferative activity of the GCs during folliculogenesis, this expression pattern is consistent with the known upregulation of RHAMM during mitosis (Sohr and Engeland, 2008) and a mitotic function of the protein in these cells.

### Activation of ERK1/2 in the *hmmr^m/m^* genetic background

The activation of ERK1/2 in granulosa cells is essential for folliculogenesis (Fan et al., 2009). Despite the absence of the HA binding motif of RHAMM in *hmmr^m/m^* MEFs, HA induced ERK1/2 phosphorylation properly (Fig. 2E). Apparently, HA stimulates ERK1/2 phosphorylation through other signalling receptors (CD44 or LYVE1 which are expressed in the ovary, are candidates) (Prevo et al., 2001) and thus remain unaffected by the RHAMM truncation. Hence absence of ERK1/2 phosphorylation could not be the cause of hypofertility. The slightly elevated HA-independent phosphorylation of ERK1/2 in *hmmr^m/m^* MEFs (Fig. 2E) has not been observed in the ovaries. Increased ERK1/2 activation is also observed in RHAMM^+/−^ ES cells (Jiang et al., 2013), in agreement with our data, but in contrast to a reported defect in ERK1/2 phosphorylation in RHAMM−/− MEFs (Tolg et al., 2006). The response to HA in the absence of the HA-binding domain, in the RHAMM C-terminus, raises the question whether RHAMM functions as surface receptor for HA at all. Indeed, this HA-binding domain is unspecific in that it can also bind heparin *in vitro* (Yang et al., 1993; Yang et al., 1994).

We therefore conclude that it is unlikely for the folliculogenesis defect of *hmmr^m/m^* mice to be due to deficiency in any interaction between HA and RHAMM. Where could the C-terminus of RHAMM be required in oocyte maturation? There are two processes where it could engage in spindle formation: meiosis of the oocytes and mitosis of the proliferating granulosa cells.

### The RHAMM C-terminus is dispensable in oocyte meiotic divisions

Oocytes gradually acquire developmental and meiotic competence during folliculogenesis, which enable the oocytes to resume meiosis. In meiosis, spindle assembly is essential for the two successive chromosome segregations to give rise to haploid oocytes (Brunet and Maro, 2005; Sánchez and Smitz, 2012). Therefore, rebuilding of the acentriolar microtubule organization center is regarded as one key feature of oocyte competence (Łuksza et al., 2013).

Rodent and human oocytes lack centrioles (Szollosi et al., 1972; Hertig and Adams, 1967). Hence acentriolar - and as a consequence anastral - spindles assemble during the meiotic divisions that conclude oogenesis. This is achieved by the chromatin-centered RanGTP gradient nucleating the mitotic spindle microtubules (Brunet and Maro, 2005; Schatten and Sun, 2011). RHAMM is involved in Ran-dependent spindle assembly *in vitro*. In *Xenopus* egg extracts, XRHAMM is essential for Ran-dependent anastral spindle assembly and it is required for spindle pole focusing via TPX2 (Groen et al., 2004). The C-terminus of XRHAMM, which encodes the centrosome targeting domain, might be sufficient for this spindle pole focusing activity (Joukov et al., 2006). Whether the microtubule-binding RHAMM N-terminus can also associate with TPX2 and function in anastral spindle focusing remains unknown. However our data suggests that it might do so. Immunofluorescence analysis demonstrated correct spindle formation and TPX2 localization at the meiotic spindles of *hmmr^m/m^* oocytes. Unfortunately analysis of RHAMM localization was inconclusive, likely due to incompatibility of the RHAMM antibody with the oocyte fixation protocol. Given that these spindles are acentrosomal, were RHAMM to localize at the mouse meiotic spindle, the microtubule-targeting domain at the N-terminus of the protein (which is intact in the *hmmr^m/m^* mouse) might suffice for this localization and for spindle pole focusing.

### The RHAMM-dependent spindle orientation in granulosa cells is required for folliculogenesis

In cell extracts and in cultured cells, two RHAMM mitotic functions have been described: maintenance of spindle integrity (Groen et al., 2004; Joukov et al., 2006; Maxwell et al., 2005) and spindle orientation (Dunsch et al., 2012).

Quantification of immature follicles in *hmmr^m/m^* ovaries at PND7 demonstrate no statistically significant differences to control *hmmr^+/+^* ovaries, albeit a potentially reduced reservoir of these follicles. What could this signify? Abnormal spindle assembly can eventually lead to chromosomal instability (Ganem et al., 2009). One can hypothesize that aberrant spindle assembly, caused by the RHAMM truncation, could drive chromosomal instability in oogonia, during embryonic *hmmr^m/m^* mouse development. Such defective oocytes are thought to be eliminated, via programmed germ cell death, in the first two post-natal days (Ghafari et al., 2007; Lobascio et al., 2007; Pepling, 2006), thereby leading to decreased number of primordial follicles by PND7.

However, previous studies indicate that RHAMM depletion is detrimental for acentrosomal spindle integrity (Groen et al., 2004; Joukov et al., 2006), but not for centrosomal spindle formation. RHAMM disruption by siRNA (Neumann et al., 2010; Dunsch et al., 2012) or a blocking antibody (Maxwell et al., 2003) in mammalian cells containing centrosomes does not prevent the majority of those cells to progress through mitosis, despite delays. Furthermore, no defects in spindle architecture of *hmmr^m/m^* granulosa cells were observed here and our quantification of *in vitro* spindle defects induced by the RHAMM C-terminus deletion (H.L., A.W. and A.P., unpublished) show them to be mild and to occur at low frequency. As such, they are unlikely to be the single contributor to the very significant folliculogenesis defects of the *hmmr^m/m^* mouse.

The second mitotic function of RHAMM, demonstrated in cultured cells, is orientation of the mitotic spindle (Dunsch et al., 2012). In human cells, this function is mediated by a central part of the RHAMM coiled coil domain, which interacts with CHICA and, via CHICA, with the molecular motor dynein to orient the mitotic spindle (Dunsch et al., 2012). The corresponding mouse RHAMM domain is not expressed in *hmmr^m/m^* (Fig. 2).

Oriented divisions, in diverse systems, can be regulated either by cell shape or by cortical cues, both of which activate dynein-dependent spindle orientation mechanisms (O'Connell and Wang, 2000; Fink et al., 2011; Kiyomitsu and Cheeseman, 2012; Xiong et al., 2014). Granulosa cells can be classified to columnar and cuboidal, according to their shape. The long axis of columnar cells is positioned perpendicular to the oocyte surface (Da Silva-Buttkus et al., 2008). This postulates that, in these cells, shape constrains can dictate spindle positioning, via dynein subcortical localization and subsequent dynein-dependent spindle anchoring. In contrast, cuboidal granulosa cells, which exhibit a five-fold higher proliferation and are located mostly adjacent to the oocyte during early follicle development divisions (Da Silva-Buttkus et al., 2008), cannot rely on shape cues for correct dynein localization and spindle orientation. This postulates the existence of a proximal-distal cortical cue in granulosa cells, enabling correct orientation of the spindle. In either case, as spindle-associated RHAMM is critical for dynein-dependent spindle orientation (Dunsch et al., 2012), the RHAMM truncation would impair spindle orientation in granulosa cells – independent of their shape. Indeed, our data confirms this function *in vivo*, demonstrating that spindle-associated RHAMM is required for spindle and division plane orientation in these cells.

This distinct orientation of the granulosa cell division plane is likely to facilitate the establishment of orderly concentric layers of granulosa cells and thus contribute to functional communication between granulosa cells and oocyte in the follicle. Such bi-directional communication is crucial for successful follicle development (Wigglesworth et al., 2013; Gilchrist et al., 2004); its impairment would be consistent with the very significant decline in number of immature and mature follicles observed in *hmmr^m/m^* ovaries.

In summary, we report the first identified physiological function for RHAMM, during oogenesis, and the importance of this function for female fertility. Our data indicate that RHAMM is a critical factor for folliculogenesis and that this spindle-associated protein is required for spindle positioning in granulosa cells, which surround the oocyte during its growth phase. Deletion of the RHAMM centrosome-targeting domain *in vivo* abolishes its spindle association, resulting in impaired spatial orientation of dividing granulosa cells, folliculogenesis defects and subsequent female hypofertility.

## MATERIALS AND METHODS

### Construction of the targeting vector and generation of the *hmmr^m/m^* mouse

The *hmmr^m/m^* mouse was generated by homologous recombination in ES cells as described previously (Reichardt et al., 1998). To delete the C-terminus (aa 318–794) of the RHAMM protein (accession number NP_038580), a targeting vector was constructed, containing the promoterless neomycin (neo)–resistance gene, as selection marker, between exon 10 and exon 11 of the full-length *hmmr*. The in-frame stop codon in the neomycin cassette resulted in premature translation stop, which gave rise to a fusion protein (predicted MW 65 kDa) comprising the RHAMM N-terminus (317 aa) and the neomycin-resistance gene product neomycin phosphotransferase II (264 aa). This targeting vector was introduced into mouse embryonic stem (ES) cells. Clones of transformed, neomycin resistant ES cells were screened for the insertion of the correct modification in the *hmmr* gene locus. A clone with a correct insertion was chosen and injected into blastocysts to generate chimeric mice. These mice were crossed with C57BL/6 to allow germline transmission of the *hmmr* mutant gene.

### Mouse colony maintenance and genotyping

The colony was maintained by breeding heterozygous animals. Backcrossing to the parental C57BL/6J strain using F1 hybrids, routinely every 10th generation, was employed to avoid production of inbred lines. Animals were provided with standard laboratory chow and tap water *ad libitum* and kept in accordance with local regulations (TLLV Thüringen, Erfurt, Germany) at constant temperature (22°C) and light cycle (12-h light, 12-h dark). Animals were sacrificed by CO_2_ inhalation. Tissues designated for western blotting analysis were snap frozen on dry ice and stored at −80°C. For *in situ* hybridization analysis, tissues were frozen in isopentane cooled on dry ice, cut into 20 µm cryo-sections on a cryostat (Leica, Wetzlar, Germany), thaw-mounted on super frost slides and stored at −80°C until further processing.

Genomic DNA was obtained from tail biopsies according to standard protocols and genotyping was performed by PCR (primers: ex11rev: 5′-TGCAGACGAGCAGACAGTTC-3′, ex10fw: 5′-AGCAAGGATAGAGAAAGGGCTG-3′, neo445rev: 5′-TGATCGACAAGACCGGCTT-3′) with an annealing temperature of 63°C, in order to discriminate between wild type *hmmr^+/+^* and mutated *hmmr^m/m^* alleles with expected sizes of 681 bp and 503 bp respectively.

### Fertility assays

Mating of 8- to 12-week-old mice, during 6 months, was used in the quantification of average number of litters. During this period, 25 litters and 115 offspring were born to five RHAMM mutant females versus 28 litters and 199 offspring to five controls.

Mating within two age cohorts, comprising 8- to 24-week-olds or mice older than 24 weeks, was used in the quantification of litter size. The offspring number for a minimum of 25 litters per genotype and age group was quantified, in total 137 litters and 831 offspring were used in the analysis.

### Culture and immortalization of mouse embryonic fibroblasts (MEFs)

Heterozygous female mice, mated with heterozygous males, were sacrificed at 14.5 days of gestation. The embryos were removed under aseptic conditions, the heads and livers were discarded and the tails were kept for genotyping. The remaining tissue was minced in 5 ml DMEM (Dulbecco's Modified Eagle Medium) supplemented with 10% FCS, 2 mM L-glutamine, 1% penicillin and streptomycin (all from Biowest) and homogenized via multiple passages through a G20 needle. The resulting cell suspension was transferred into a 10 cm-diameter tissue culture-grade Petri dish and incubated at 37°C in 5% CO_2_. The medium was changed and unattached cells were removed over the following days. When the attached “primary MEFs”, which were designated as passage number 0, reached confluency, they were subcultured every 3 days at a density of 20,000 cells/cm^2^. After 6–10 passages the primary MEFs exhibited growth arrest. Approximately 20 passages after this point, they regained the ability to grow. The resulting cells are designated as “immortalized MEFs”. Both primary and immortalized MEFs were mycoplasma-negative.

### Histological analysis, ovarian follicle quantification, orientation of granulosa cell division

Mouse ovaries and uterus were fixed with 4% paraformaldehyde for 16–24 h and embedded in paraffin. 4 µm thick tissue sections were stained with hematoxylin and eosin according to standard protocol. The sections were analyzed in an Olympus BX41 light microscope and images were acquired using the Cell* software (Olympus).

For ovarian follicle quantification, 4 µm thick serial sections of the whole ovary were prepared and one of every five sections was stained with hematoxylin and eosin. The sections were examined with bright field microscopy, the follicles, at different stages of folliculogenesis, were counted and the total number of follicles in the sections examined were plotted (see Figs 3 and 4) (Canning et al., 2003; Tilly, 2003).

For the morphological classification of the follicles, the criteria of Pedersen and Peters (Pedersen and Peters, 1968) were applied. Briefly: follicles type 1,2 and 3 were classified as primordial; type 4 and 5 were classified as primary; type 6 as secondary; type 7 as antral and type 8 as pre-ovulatory.

For analysis of the orientation of granulosa cell division, ovary sections, prepared as described above, were scanned with 40× objective in a VS110 virtual microscope (Olympus). From the scanned images, follicles with one or two layers of granulosa cells were selected and the cell division axis of granulosa cells at metaphase or anaphase, was determined: A line dissecting the center of the oocyte, the centre of the mitotic granulosa cell and the basal lamina was defined as the oocyte-basal membrane axis (Fig. 5G, orange line). For cells at anaphase, the spindle axis was defined as the line parallel to the direction of separating chromosomes (Fig. 5G, black line). For cells at metaphase, a line along the metaphase plane was drawn (Fig. 5G, red line) and a second line perpendicular to the metaphase plate (Fig. 5G, black line) was used to define the spindle axis. The angle θ between oocyte-basal membrane axis and spindle axis was determined using ImageJ (NIH). The follicles of 4 wild type mice ovaries (n = 41 mitotic cells) and 8 mutant mice ovaries (n = 31 mitotic cells) were thus analyzed.

### Mouse oocyte collection and *in vitro* maturation

Oocyte *in vitro* maturation assays were performed as previously described (Brunet et al., 2008). Briefly, oocytes were collected from ovaries of 10- or 26-week-old *hmmr^+/+^* and *hmmr^m/m^* mice and placed in M2 medium pre-warmed to 37°C and supplemented with 4 mg/ml BSA and 1 mM milrinone. For video microscopy of oocyte meiotic maturation, oocytes were transferred to a Ludin Chamber containing M2 medium with 4 mg/ml BSA. Time-lapse images were acquired using a Photometrics CCD camera (CoolSnap HQ2) mounted on a Leica HC PL APO 20×/0.7 NA objective enclosed in a thermostatic chamber (Life Imaging Service). Images were taken every 15 min for 18–20 h at 20× magnification. Metamorph 7.0 (Universal Imaging) and ImageJ (NIH) software were used for image analysis.

### RT-PCR

Total RNA was extracted from MEFs and reverse transcribed into cDNA using the High Fidelity cDNA Synthesis kit (Roche). Amplification of the indicated regions of RHAMM was performed with the respective primer pairs listed below. GAPDH was used as control.

hmmr exons 1–5: frw: 5′-GACCCTTCGGGTTGTGCTCCATC-3′; rev: 5′-GCCTTTAGTAGCTCGTTGGCTCTGG-3′

hmmr exons 7–12: frw: 5′-GGTCAAACAGGAAGGCATGGAGC-3′; rev: 5′- CTCACGCCCAAGCCATCTTGA-3′

hmmr exons 18–21: frw: 5′-AAGGCAACCCAAACTGCTGCTG-3′; rev: 5′-CCCTTGTGGTTGGTGCTGTCTC-3′

### *In situ* hybridization

*In situ* hybridization was performed as described previously (Trajkovic-Arsic et al., 2010). Fresh-frozen sections from wild type ovaries and uterus were hybridized with S35-labeled riboprobes corresponding to nucleotides 1–540 of mouse RHAMM mRNA (accession number NM_013552). Following post-hybridization, the sections were covered with photo-emulsion (NTB, Kodak) and stored at 4°C. Autoradiograms were analyzed and images were acquired under dark-field illumination. Control hybridization experiments employing a sense probe did not reveal any specific hybridization signal.

### Antibodies

A synthetic peptide corresponding to aa1-241 of RHAMM (accession number: NP_038580) was used to immunize rabbits; the resulting anti-RHAMM polyclonal antibody was affinity-purified from rabbit serum. The primary antibodies, used in immunofluorescence (IF) and western blotting (WB) experiments, are shown in Table 1. Immunoaffinity-purified Alexa-conjugated goat or HRP-conjugated donkey secondary antibodies were used in the dilutions indicated: Alexa-fluor-488-conjugated (1/300) or -594-conjugated (1/400) (Molecular Probes, Invitrogen); HRP-conjugated (1/5000) (Jackson Immunoresearch Laboratories). The blocking buffers indicated (immunolabelling, western blotting) were used as primary and secondary antibody diluents.

**Table 1.**
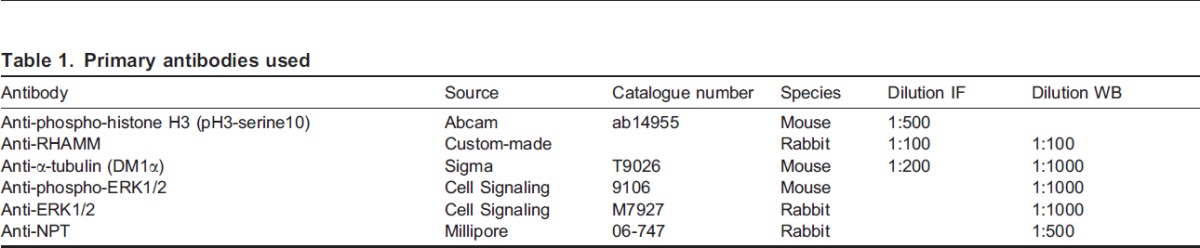
Primary antibodies used

### Immunolabelling

Paraffin-embedded ovary sections were deparaffinized and rehydrated. For heat-mediated antigen retrieval, the tissue sections were placed in citrate buffer (10 mM, pH 6.0) and incubated in a pressure cooker at 125°C for 20 min. The sections were allowed to cool-down for 30 min, washed in PBS, incubated with immunohistochemistry blocking buffer (5% BSA, 5% goat serum, 0.1% triton X-100) for 1 h, followed by primary antibody incubation at 4°C overnight. The samples were washed in PBS (10 min, 3 changes) and incubated with Alexa-conjugated secondary antibodies for 1 h, followed by counter-staining of nuclei with 1 µg/ml DAPI (4′,6-diamidino-2-phenylindole, Sigma) for 3 min. All steps were performed at room temperature, unless otherwise indicated.

Images were acquired on an Axiovert200 microscope equipped with a 12-bit grayscale cooled CCD AxioCamMRm camera (Zeiss). Representative images were brought to a resolution of 300 ppi without re-sampling using Adobe Photoshop (Adobe) and the area of interest was cropped.

### ERK1/2 activation assay

MEFs were incubated with serum-free medium for 2 h at 37°C. They were subsequently stimulated for 15 min with the addition of 100 µg/ml high-molecular-weight hyaluronic acid or 10% serum in the medium and processed for immunoblotting.

### Preparation of cell protein lysates

Cells were harvested by “scraping” into ice-cold PBS containing protease and phosphatase inhibitors (10 mM NaV, 50 mM NaF, 50 mM β-glycerophosphate, 10 mM PMSF 10 µg/ml antipain, 10 µg/ml chymostatin, 1 µg/ml pepstatin A, 2 µg/ml leupeptin, 200 µg/ml pefabloc/AEBSF-HCl, 2 µg/ml aprotinin) and centrifugation (100 ***g***, 5 min, 4°C). The supernatant was discarded and one pellet volume of 2× Laemmli sample buffer (4% w/v SDS, 20% glycerol, 0.2% w/v bromophenol blue, 156 mM β-mercaptoethanol, 100 mM Tris-HCl pH 6.8) was added. Lysate proteins were denatured at 100°C for 5 min, the suspension was sonicated to shear DNA and stored at −80°C.

### SDS-PAGE and western blotting

Proteins were separated by SDS-PAGE using 10% acrylamide gel in running buffer (25 mM Tris, 250 mM glycine, 0.1% (w/v) SDS). Proteins were transferred onto nitrocellulose membrane (0.2 µm pore size, PROTRAN, Whatman) using a wet transfer system (Mini-Protean, BioRad) at 30 V, 4°C, overnight in transfer buffer [50 mM Tris, 380 mM glycine, 0.05% (w/v) SDS, 20% (v/v) methanol). Membranes were stained with Ponceau S solution (0.25% (w/v) Ponceau S (Serva), 40% methanol (Merck), 15% acetic acid (Roth) in H_2_O] for total protein visualization, briefly washed and then incubated for 30 min in WB blocking buffer (5% non-fat milk, 0.1% Tween-20 in PBS). Membranes were incubated with primary antibody diluted in blocking buffer for 1 h, washed four times with 0.1% Tween-20 in PBS, incubated in secondary, HRP-conjugated, antibody for 45 min and washed as described above. Antibody labelling was visualised by chemiluminescence using the western lightning plus-ECL Kit (Perkin Elmer) and detected on BioMax MR film (Kodak).

### Statistical analysis

The fertility assays (Fig. 2) were analyzed by the two tail Student's t-test, the *in vitro* oocyte maturation assays (supplementary material Fig. S1) by the Fisher's Exact test, the granulosa cell spindle angle quantification (Fig. 5) by the Mann-Whitney test. Results are presented as mean±s.d., with error bars denoting the standard deviation. The hypothesis that spindle orientation in granulosa cells is random (Fig. 5) was tested by the Kolmogorov-Smirnov distribution test, applied on the distribution of spindle angles in granulosa cells versus a random distribution, normalising the angle θ (Fig. 5) between 0 and 1 (D = 0.1876, p = 0.6416 for *hmmr^m/m^* ; D = 0.5582, p = 1.601×10^−11^ for *hmmr^+/+^*).

## Supplementary Material

Supplementary Material
