# Supplementary material for: RHAMM deficiency disrupts folliculogenesis resulting in female hypofertility
Source: Biol Open. 2015 Mar 6;4(4):562–71. doi: 10.1242/bio.201410892 (PMC4400598; doi:10.1242/bio.201410892)
Supplement: Supplementary Material [file supp_4_4_562__index.html]

RHAMM deficiency disrupts folliculogenesis resulting in female hypofertility — RHAMM deficiency disrupts folliculogenesis resulting in female hypofertility — Supplementary Material 

# RHAMM deficiency disrupts folliculogenesis resulting in female hypofertility

## bio.201410892 Supplementary Material

**Files in this Data Supplement:**

- Supplementary Material - Huaibiao Li et al. doi: 10.1242/bio.201410892
