# Supplementary material for: RHAMM deficiency disrupts folliculogenesis resulting in female hypofertility
Source: Biol Open. 2015 Mar 6;4(4):562–71. doi: 10.1242/bio.201410892 (PMC4400598; doi:10.1242/bio.201410892)
Supplement: Supplementary Material [file supp_bio.201410892_bio.201410892-s1.pdf]

Biology Open (2015) 000, 1–10 doi:10.1242/bio.201410892

Supplementary Material
Huaibiao Li et al. doi: 10.1242/bio.201410892

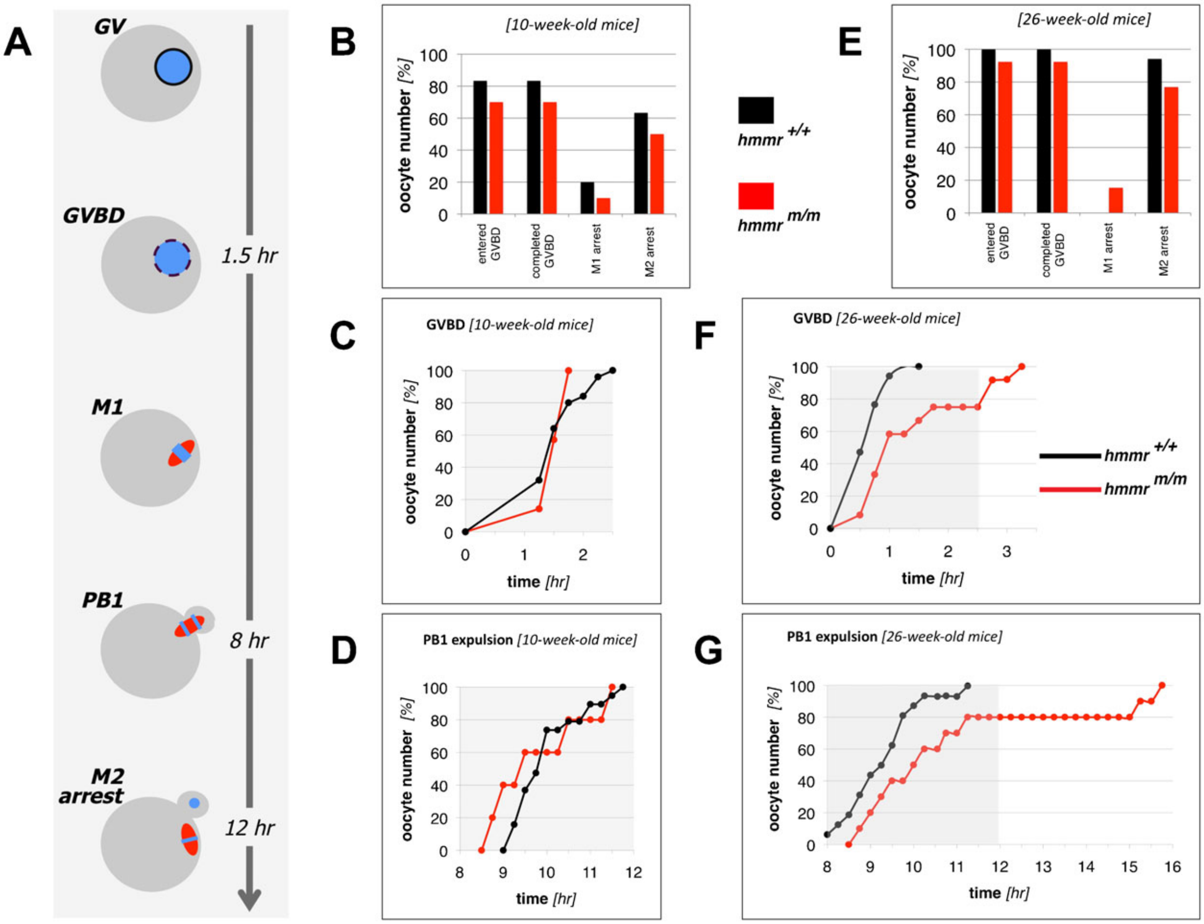

Fig. S1. RHAMM deficiency does not impair oocyte meiotic maturation. (A) Schematic overview of the oocyte meiotic maturation, indicating the different stages and their average duration. GV: germinal vesicle, GVBD: germinal vesicle breakdown, M1: meiosis I, PB1: expulsion of the 1st polar body, M2: meiosis II; blue colour: chromatin, red colour: microtubules. (B–G) Oocyte maturation was analyzed via video microscopy, for oocytes of 10 week-old (B–D) and 26 week-old (E–G) wild type and *hmmr*$^{m/m}$ mutant mice. The number of oocytes at GVBD, M1- or M2-arrested are plotted as cumulative percent. Abbreviations as in A. Despite the reduction in total number of oocytes in *hmmr*$^{m/m}$ mutants, oocyte *in vitro* maturation assays indicate no defects in oocyte maturation except for a significant delay ($p<0.05$) in GVBD of the older *hmmr*$^{m/m}$ oocytes (F).

Biology Open
